# A multi-model prediction of a stage-specific prognosis for colorectal cancer using attention-driven deep ensemble learning on genomic profiling data

**DOI:** 10.3389/frai.2026.1856972

**Published:** 2026-07-21

**Authors:** K. Supriya, A. Anitha

**Affiliations:** School of Computer Science Engineering and Information Systems, Vellore Institute of technology, Vellore, India

**Keywords:** attention mechanism, Bi-LSTM, colorectal cancer prediction, deep ensemble learning, genomic profiling, rough set, survival analysis, WOA

## Abstract

**Introduction:**

Over the decades, shifts in human lifestyle have led to alterations in dietary habits. The consumption of diets low in fiber and high in fat and sugar results in the production of carcinogenic metabolites during digestion. The food we consume undergoes a complex series of processes involving digestion and excretion, engaging various internal organs within the human body. The DNA and MiRNA present in food are crucial for sustaining human health. Damage to human organs can lead to the development of cancer cells. Among various cancers, Colorectal Cancer (CRC) is the 3rd most common cancer that contributes to the increase in the mortality rate worldwide.

**Methods:**

One of the better ways for early CRC diagnosis is through Genomic Profiling. Frequent mutations in the genes APC, TP53, KRAS, PIK3CA, and SMAD4 are observed in the collected samples, contributing to CRC. An effort has been made in the proposed work to improve classification and prediction by using a deep ensemble learning approach based on a self-attention-based stacked bidirectional LSTM, optimized with the Parallel-Whale Optimization Algorithm (WOA) for global convergence, following a feature selection process. Furthermore, the survival analysis of CRC patients is performed using the DeepSurv technique to assess treatment effectiveness and patient care.

**Results:**

The performance of the proposed model is evaluated using various metrics for stage-based colorectal cancer prediction, and a comparative analysis is conducted to validate against benchmarking techniques, resulting in improved classification and prediction accuracy.

**Discussion:**

This study may help physicians detect CRC earlier and improve patient management.

## 1 Introduction

Around the world, Colorectal cancer (CRC) is the third most common cancer and remains a major contributor to global cancer morbidity and mortality (Qwaider et al., 2021). Colon adenocarcinoma (COAD) and Rectal adenocarcinoma (READ) are two common types of colon cancer that usually arise when the epithelial cells lining the colon undergo aberrant changes that allow malignant cells to invade and grow. The survival probability for colon cancer is 90% if it is discovered in the early stages (Chang et al., 2021; Zou et al., 2022). The progression of CRC is influenced by genetic alterations, which are also seen in other forms of cancer (Tao et al., 2024; Lei and Tao, 2020). Advanced molecular integrative analyses have revealed that distinct protein-level alterations and associated tumor suppressor genes, such as APC, KRAS, TP53, PIK3CA, and SMAD4, are major contributors to tumorigenesis (Noack and Langer, 2023; Feliu et al., 2024; Jin et al., 2024; Osakabe et al., 2025). With recent advances in gene-sequencing platforms and network databases, such as “The Cancer Genome Atlas (TCGA)” and “The Gene Expression Omnibus (GEO),” it has become possible to identify the genes that play a role in carcinogenesis (Zabeti Touchaei et al., 2024; Chang et al., 2020). The TCGA repository contains large amounts of data; however, not all of it may be useful for prediction. Simultaneously, ignoring some data leads to a wrong prediction. The genomic mutations in APC, KRAS, PIK3CA, and SMAD4 can be interpreted as ordered vectors where upstream and downstream signals jointly influence CRC progression. Thus, it is important to identify a model that can extract significant features from large amounts of data and streamline the process for better classification and prediction.

In recent years, the medical field has extensively integrated protein-centric insights into various Machine Learning (ML) and Deep Learning (DL) methods to improve disease prediction. An effort has been made in this study to identify the significant features by applying rough set-based core and reduct. The consistent data is then applied to deep ensemble learning using a self-attention-based stacked Bi-LSTM to capture complex sequential patterns and focus on the most relevant parts of the input, with optimal parameters for the classification and prediction of CRC. A Bidirectional LSTM was adopted to capture both forward and backward contextual dependencies, enabling the model to learn how combinations of mutations contribute to stage-specific outcomes. The choice to use Bi-LSTM enhances predictive accuracy while maintaining interpretability by leveraging attention mechanisms that emphasize meaningful signals such as APC, KRAS, PIK3CA, and SMAD4 mutations. To obtain the optimal hyperparameters, the metaheuristic Parallel-Whale Optimization Algorithm (WOA), which balances exploration and exploitation for global convergence, is used. Furthermore, survival analysis was performed with Deepsurv, using the Cox proportional hazards model for treatment and patient care.

This study is articulated as follows: Section 2 discusses previous research on feature selection techniques and deep learning prediction processes, thereby providing the background fundamentals, as in Section 3. The empirical study on the proposed research model, as Section 4 explains, data preprocessing, feature engineering, and the Whale optimization algorithm to ensure Global convergence. Section 5 discussed experimental analysis and results for stage-based CRC prediction, followed by a comparative analysis with benchmarked models, which is discussed in Section 6. Finally, Section 7 discussed the survival analysis of patients affected with protein changes in influential genes of CRC with a case study. The survival analysis was carried out using the DeepSurv model, followed by a conclusion and future enhancement of the proposed research.

## 2 Related works

This section presents related studies across various research directions on the prediction and survival processes of colorectal cancer.

### 2.1 Related studies on the feature engineering process

Neural networks can learn relationships and recognize patterns effectively if the training data is consistent. However, real-world clinical and genomic data are inconsistent and have missing values. To establish consistency in the data, various imputation methodologies have been discussed by researchers. Fan et al. (2024) used a miss forest imputation technique to fill missing values in multivariate epigenome-wide-DNA methylation data of colorectal cancer by achieving high R-squared *R*^2^ and low Root Mean Square Error (RMSE) and Mean Absolute Error (MAE). Pawlak (1996) and Xu and Zhao (2025) utilized an attribute approximation algorithm based on a neighborhood variable-precision rough set to reduce the number of attributes in oncogene data and achieved better diagnostic accuracy. Askr et al. (2025) applied a fuzzy rough set feature selection technique to extract the significant chemical compound features from the investigated data to identify a potential new drug for skin cancer. Bhukya and Sadanandam (2023) employed a rough set-based quick reduction technique to reduce feature dimensionality for breast cancer, predicting cancerous breast nodules using the Wisconsin dataset. Amrir et al. (2026) utilized the Whale optimization algorithm for tuning hyperparameters with a hybrid-LSTM model on a lung cancer dataset and obtained better optimal parameters with better accuracy. Yalçiner (2025) utilized an AI-augmented deep learning model for survival prediction in *de novo* metastatic colorectal cancer, employing the AdamW optimizer and achieving better results.

The following are the merits and demerits of this subsection.

**Merits:**

- Dimensionality reduction helps in effective computation.
- Normalization helps maintain consistency and a uniform contribution across the dataset.

**Demerits:**

- The use of imputation methods can introduce bias in the randomized missing data, causing potential data distortion.
- Data normalization and feature selection methods such as chi-square tests, ANOVA, and PCA may oversimplify the data, potentially failing to capture non-linear relationships that are critical for achieving optimal predictive performance.

### 2.2 Related studies on prediction of colorectal cancer with deep learning

Pateriya et al. (2025) developed a non-invasive AI-ML tool called CRCpred that predicts colorectal cancer using gut microbiome profiles and demonstrated better accuracy using XGBoost, and classified the health status of healthy individuals and CRC patients. Wang et al. (2025) employed a deep learning model to predict the binding affinity between drugs and their corresponding protein targets associated with Crohn's disease from druggable genes. Shah et al. (2025) employed a hybrid deep learning model based on a Laplacian score-CNN for classification of microarray cancer data and achieved higher accuracy. Mulenga et al. (2025) utilized a deep neural network (DNN) to detect colorectal cancer from gut microbiome data and achieved robust analysis using six normalization and ranking techniques. Sethi et al. (2023) developed an LSTM-Deep Belief Network model for classification using microarray gene expression data and achieved improved predictive performance. Mahmoud et al. (2024) developed an LSTM-based cancer prediction model for classification on the Breast Cancer dataset, achieving better accuracy for early-stage survival prediction using gene data. Fan and Xu (2024) developed a Bi-LSTM using self-Attention mechanisms, based on a weighted fusion method applied to protein data, to predict functional changes in protein mutations and achieved improved predictive performance. Gou et al. (2025) employed a Geometric Vector Perceptron-Graph Neural Network to learn structure-aware protein representations and successfully classified proteins into four origin categories using structural data. Supriya and Anitha (2024a,b) utilized DeepSurv and Cox models for survival analysis of bladder cancer patients and for stage-based colorectal cancer prediction on uncertain data, using rough computing and LSTM models to obtain better results.

**Merits:**

- Machine learning models are capable of handling limited features, making them useful in clinical research that uses fewer genetic markers.
- Deep learning models can effectively combine and analyze distinct types of biological data, such as genomic and proteomic data.

**Demerits:**

- The prediction of the deep learning models suffers from insufficient explainability.
- The obtained results lack clinical validation.
- The computation time increases as the amount of data increases.

**From the above studies, the following research gaps are identified:-**

- Most studies rely on small, imbalanced, and single-centered data with uncertainty.
- Limited exploration of feature importance and biomarker explanation on gene expression data.
- Lack of a standardized framework for pre-processing the uncertain data, and the challenge in identifying a suitable neural network architecture to obtain a better prediction model.

**Thus, the primary objectives are:-**

- To obtain potential knowledge by employing **rough set-based core and reduct algorithm** among the attributes to **maintain data consistency**.
- The consistent data is fed into a **deep ensemble learning** framework optimized with the Whale optimization algorithm (WOA) to improve the global convergence rate, thereby obtaining optimal parameters for classifying and predicting colorectal cancer stages using previously **unseen data**.
- A self-attention mechanism is incorporated to dynamically weight the hidden states, enabling the model to identify weak learners and improve its predictive capability.
- The **progression of cancer stages** is explored by identifying **influential genes** and analyzing their corresponding gene mutations.
- To perform **Survival analysis** using **Cox Proportional-hazard** via **DeepSurv technique** to estimate the patient-specific risk on the identified genomic markers.

## 3 Background fundamentals

In recent years, extracting meaningful information from real-world datasets is more challenging. The collected datasets are characterized by noise, redundancy, and inconsistencies. Therefore, a comprehensive understanding of the underlying information system is essential. In this study, the collected data contain conflicting and irrelevant information, leading to data inconsistency. To mitigate these issues, the core and reduct concepts of rough set theory were applied using an information system. This section presents the fundamentals of core and reduct in rough set theory, along with the basic principles of self-attention–based stacked Bi-LSTM techniques.

### 3.1 Roughset-core and reduct

The real-world data collected from a particular environment may contain uncertainty, that is, data may be imprecise or incomplete. To address uncertainty, Pawlak (1996) introduced rough set theory, which offers a novel and powerful approach to handling vagueness and uncertainty. Before discussing rough set theory, it is important to understand an information system. An information system is a table in which every column represents an attribute, whereas the row represents an object.

More formally, an information system is a quadruple *IS* = (*U, A*, Val, *f*) where *U* = {*p*_1_, *p*_2_, *p*_3_…*p*_*n*_} is a non empty finite set of objects called universe. *A* = {*a*_1_, *a*_2_, *a*_3_…*a*_*n*_} be as finite set of attributes. For every *a* ∈ *A*;, Val = ⋃_*a* ∈ *A*_Val_*a*_, where Val_*a*_ represents the attribute *a* ∈ *A*. *f*_*a*_:*U* × *A* → Val_*a*_, is the information function such that *f*(*p, a*) ∈ Val_*a*_ for every *a* ∈ *A*, *p* ∈ *U*. Further if *CA, DA* ⊆ *A*, such that *CA* ∪ *DA* = *A* and *CA* ∩ *DA* = ϕ, there the information system is known as decision system., where *CA* is the conditional attributes and *DA* is the set of decisions (Pawlak, 1996). For example, consider a sample decision system presented in Table 1. In the above table, *U* = {*p*_1_, *p*_2_, *p*_3_…*p*_8_}, *A* = {*a*_1_, *a*_2_, *a*_3_} the value of the attribute {*a*_1_} is given as Val*a*_1_ = {Senior, Adult, Young}. Similarly, we have others, and Stage *(d)* is represented as a decision attribute.

**Table 1:** Information system.

Patients (objects)	Age (*a*_1_)	CEA (*a*_2_) (ng/ml)	KRAS (*a*_3_)	Stage (*d*)
*p*_1_	Senior	High	Mutated	3
*p*_2_	Adult	Medium	Mutated	2
*p*_3_	Young	Low	Wild-type	1
*p*_4_	Senior	High	Mutated	4
*p*_5_	Senior	Low	Mutated	2
*p*_6_	Young	Low	Wild-type	1
*p*_7_	Senior	High	Mutated	4
*p*_8_	Adult	Low	Mutated	2

#### 3.1.1 Indiscernibility relation

The indiscernibility relation is a core concept in rough set theory. It represents a relationship among two or more objects that share identical values for a given set of attributes, as described by Pawlak (1991). The indiscernibility relation is formally defined as follows.

Let *P* ⊆ *A*. The equivalence relation *ER*(*P*) on *U* is given in Equation 1.


$$\begin{array}{c} ER\left(P\right)=\left\{\left(p_{i},p_{j}\right);a\left(p_{i}\right)=a\left(p_{j}\right)\;\forall a\in P\right\} \end{array}$$ (1)

The equivalence class of *U* induced by *P* is given as *ER*(*P*) or *U*/*P*. For example, consider the decision in Table 1; the indiscernibility relation can be generated as follows from Equations 2–5.


$$\begin{array}{c} U/\left(A=\left\{a_{1}\right\}\right)=\left\{\left\{p_{1},p_{4},p_{5},p_{7}\right\},\left\{p_{2},p_{8}\right\},\left\{p_{3},p_{6}\right\}\right\} \end{array}$$ (2)

$$\begin{array}{c} U/\left(A=\left\{a_{2}\right\}\right)=\left\{\left\{p_{1},p_{4},p_{7}\right\},\left\{p_{2}\right\},\left\{p_{3},p_{5},p_{6},p_{8}\right\}\right\} \end{array}$$ (3)

$$\begin{array}{c} U/\left(A=\left\{a_{3}\right\}\right)=\left\{\left\{p_{1},p_{2},p_{4},p_{5},p_{7},p_{8}\right\},\left\{p_{3},p_{6}\right\}\right\} \end{array}$$ (4)

$$\begin{array}{c} U/\left(A=\left\{a_{1},a_{2},a_{3}\right\}\right)=\left\{\left\{p_{1},p_{4},p_{7}\right\},\left\{p_{2}\right\},\left\{p_{3},p_{6}\right\},\left\{p_{5}\right\},\left\{p_{8}\right\}\right\} \end{array}$$ (5)

It is seen clearly from the above analysis that an equivalence relation is an indiscernibility relation over the set *U*. Thus, it divides the set of objects into disjoint equivalence classes. Therefore, for the given set of conditional attributes *CA*, each equivalence class contains the set of indiscernible objects.

The indiscernibility is obtained for each object *p*_*j*_, *j* = 1, 2, ...8, and is compared against each other object (cell-wise) for the set of attributes considered. Foe example, given the attribute Age (*a*_1_) the objects {{*p*_1_, *p*_4_, *p*_5_, *p*_7_} are indiscernible (similar). Likewise the objects {*p*_2_, *p*_8_} and {*p*_3_, *p*_6_} are indiscernible. Similarly, for the attribute CEA {*a*_2_} and KRAS {*a*_3_}, the indiscernible objects are given in Equations 3, 4. The indiscernibility matrix for the information system under consideration is given in Table 2.

**Table 2:** Indiscernibility matrix for information system *(IS)*.

Patients	(*p*_1_)	(*p*_2_)	(*p*_3_)	(*p*_4_)	(*p*_5_)	(*p*_6_)	(*p*_7_)	(*p*_8_)
*p*_1_	-	-	-	-	-	-	-	-
*p*_2_	{*a*_3_}	-	-	-	-	-	-	-
*p*_3_	{ϕ}	{ϕ}	-	-	-	-	-	-
*p*_4_	{*a*_1_, *a*_2_, *a*_3_}	{*a*_3_}	{ϕ}	-	-	-	-	-
*p*_5_	{*a*_1_, *a*_3_}	{*a*_3_}	{*a*_2_}	{*a*_1_, *a*_3_}	-	-	-	-
*p*_6_	{ϕ}	{ϕ}	{*a*_1_, *a*_2_, *a*_3_}	{ϕ}	{*a*_2_}	-	-	-
*p*_7_	{*a*_1_, *a*_2_, *a*_3_}	{*a*_3_}	{ϕ}	{*a*_1_, *a*_2_, *a*_3_}	{*a*_1_, *a*_3_}	{ϕ}	-	-
*p*_8_	{*a*_3_}	{*a*_1_, *a*_3_}	{*a*_2_}	{*a*_3_}	{*a*_2_, *a*_3_}	{*a*_2_}	{*a*_3_}	-

#### 3.1.2 Core and reduct

The main objectives of rough set theory are feature reduction and the identification of core attributes. If the classification power achieved by considering the complete set of attributes can be preserved by a minimal subset within the information system, such a subset is called a reduct. A reduct reduces computational complexity when maintaining the same classification performance as the full attribute set. The intersection of all reducts is used to determine the core attribute(s). In real-world applications, reducts eliminate redundant attributes and retain only essential specific classification outcomes generated by attributes *P*, where *P* ⊆ *A*, thus providing the decision-maker with simplified and relevant information. Based on the dependency properties of attributes, when an attribute set is dependent, the process initiates identifying all possible minimal subsets that produce the same number of elementary sets (Walczak and Massart, 1999). To formally prove the above equations, taking the help of some auxiliary notations is necessary: Let *P* ⊆ *A*, and for every *a* ∈ *P*, the attribute *a* is considered dispensable in *P* if the Equation 6 is satisfied; otherwise, *a* is considered indispensable in *P*.


$$\begin{array}{c} U/P=U/\left(P-\left\{a\right\}\right) \end{array}$$ (6)

If all the attributes are indispensable in set *P*, then *P* is said to be independent. A Reduct *P*′ of *P* is represented as *P*′ ⊆ *P*, such that the equivalence class induced by the attribute set *P*, i.e., *U*/*P* = *U*/*P*′. The core of the attribute set *P* consists of all indispensable (necessary) attributes of *P* (Pawlak, 1996). An important property linking the concept of core and reducts is presented in Equation 7, where Reduct(*P*) denotes the set of all reducts (subsets) of *P*.


$$\begin{array}{c} \text{Core}\left(P\right)=⋂\text{Reduct}\left(P\right) \end{array}$$ (7)

The core attribute is the most essential feature of knowledge that cannot be eliminated when extracting knowledge from an information system. To illustrate this concept, consider the information system in Table 3. Hence, we get the steps followed:

**Table 3:** Qualitative information system.

Patients (Objects)	KRAS(*a*_1_)	CEA (*a*_2_) (ng/ml)	Age (*a*_3_)	Stage (*d*)
*p*_1_	High	High	Mutated	High
*p*_2_	Medium	Medium	Mutated	Medium
*p*_3_	Low	Low	Wild-type	Low
*p*_4_	High	High	Mutated	Very high
*p*_5_	High	Low	Mutated	Medium
*p*_6_	Low	Low	Wild-type	Low
*p*_7_	High	High	Mutated	Very high
*p*_8_	Medium	Low	Mutated	Medium

1. As an initial step, the indiscernibility relations are computed for all the attributes as follows.


$$\begin{array}{c} U/\left(a_{1},a_{2}\right)=\left\{\left\{p_{1},p_{4},p_{7}\right\},\left\{p_{2}\right\},\left\{p_{3},p_{6}\right\},\left\{p_{5}\right\},\left\{p_{8}\right\}\right\} \end{array}$$

$$\begin{array}{c} U/\left(a_{2},a_{3}\right)=\{\left\{p_{2}\right\},\left\{\left\{p_{1},p_{4},p_{7}\right\},\left\{p_{3},p_{6}\right\},\left\{p_{5},p_{8}\right\}\right\} \end{array}$$

$$\begin{array}{c} U/\left(a_{1},a_{3}\right)=\left\{\left\{p_{1},p_{4},p_{5},p_{7}\right\},\left\{p_{2},p_{8}\right\},\left\{p_{3},p_{6}\right\}\right\} \end{array}$$

$$\begin{array}{c} U/\left(a_{1},a_{2},a_{3}\right)=\left\{\left\{p_{1},p_{4},p_{7}\right\},\left\{p_{2}\right\},\left\{p_{3},p_{6}\right\},\left\{p_{5}\right\},\left\{p_{8}\right\}\right\} \end{array}$$

2. From the above equation analysis, it is clear that the observed indiscernible attributes are *U*/(*a*_1_, *a*_2_) = *U*/{*a*_1_, *a*_2_, *a*_3_}. Therefore, the attribute *a*_3_ is dispensable. Similarly, *U*/(*a*_2_, *a*_3_) ≠ *U*/{*a*_1_, *a*_2_, *a*_3_} and hence *a*_1_ is indispensable. Also, *U*/(*a*_1_, *a*_3_) ≠ *U*/(*a*_1_, *a*_2_, *a*_3_) hence *a*_2_ is indispensable.

3. On considering the single attributes *U*/(*a*_1_, *a*_2_) ≠ *U*/(*a*_1_) and *U*/(*a*_1_, *a*_2_) ≠ *U*/(*a*_2_). Therefore, the only reduct is given as *Reduct*(*P*) = {*a*_1_, *a*_2_}.

4. Generally, the core is the intersection of all the reducts, but the above information system has only one reduct, which acts as the core. Thus *Core*(*A*) = {*a*_1_, *a*_2_}.

Therefore the core attributes are {*a*_1_} = Age and the attribute {*a*_2_} = CEA.

An information system can have many reducts, and the intersection of these reducts helps identify significant attributes. Thus, rough set-based core and reducts help in maintaining data consistency by identifying the significant attributes.

### 3.2 Basics of self-attention-based stacked Bi-LSTM

**Figure 1:**
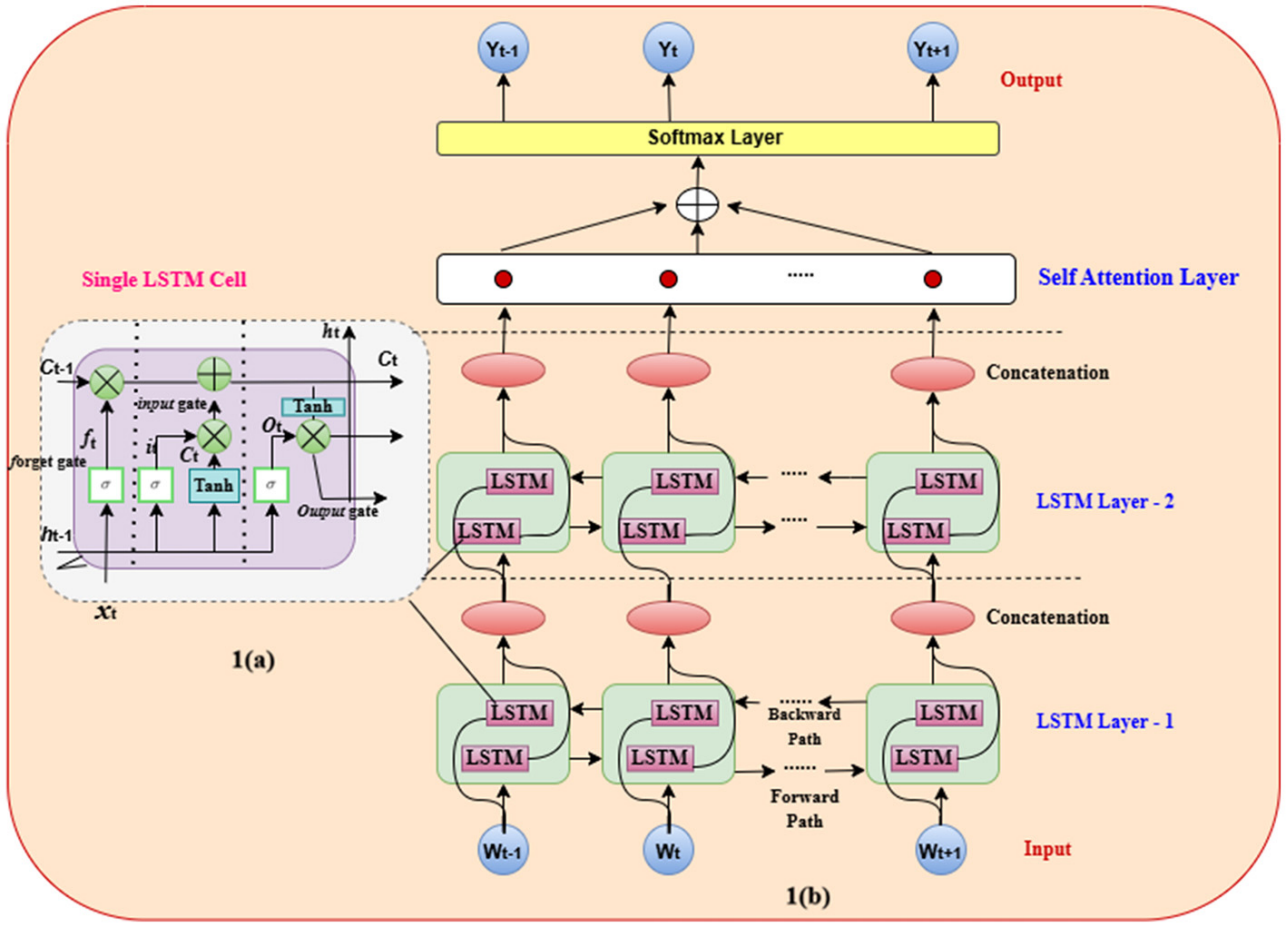
Structure of stacked two-layered self-attention-based bidirectional LSTM. **(a)** Single LSTM cell. **(b)** Stacked Bi-LSTM architecture.

A Stacked Bidirectional LSTM (SBi-LSTM) is a deep neural network architecture that stacks multiple Bidirectional LSTM layers, enabling the model to effectively process sequential data. Each layer captures both forward and backward contextual information, allowing comprehensive integration of past and future dependencies within the sequence. The structure of single LSTM cell is represented in Figure 1a and SBi-LSTM is shown in Figure 1b.

In Figure 1a, a single-cell unidirectional LSTM unit consists of feed-forward neural networks that contain three gates (input gate, forget gate, and output gate). The three gates support in regulating the information flow and long-term dependencies. The gradient descent issues faced by recurrent neural networks (RNN's) are avoided by LSTM. *W*_*t*_ represents the input vector at timestamp *t*; the retention of long-term dependencies is handled by memory cells *C*_*t*_, *C*_*t*−1_ at times step *t* and *t* − 1. The short-term memories are handled by hidden states *h*_*t*_ and *h*_*t*−1_ at time step *t* and *t* − 1. A (σ) function is a non-linear activation function, and the *tanh* function for short-term memory is used in LSTM gates to support the network in determining the data to retain and discard.

In each cell, at every time step (*t*), the Input gate (*i*_*t*_) controls the integration of recent data, and the Forget Gate (*f*_*t*_) permits the cell state to forget the previous memory. Moreover, the Output Gate (*Y*_*t*_) controls the cell state output and shifts to the next time step. In Figure 1b, the Bi-directional LSTM updates the cell states using gating mechanisms while processing sequences in both forward and backward directions to capture contextual information. A stacked Bi-LSTM consists of multiple Bi-LSTM layers, with each layer receiving the output of the previous layer. However, in long sequences, important information may become diluted. Therefore, a self-attention mechanism assigns adaptive weights to emphasize the most informative hidden states.

The process of the self-attention mechanism begins with an input sequence represented as *X* = {*x*_1_, *x*_2_, …, *x*_*T*_*Seq*__}, where *T*_*Seq*_ denotes the sequence length, and each feature vector $x_{t}\in {\mathbb{R}}^{d}$ has dimension *d*. This sequence is processed by a Bi-LSTM network to capture contextual dependencies in both the past and future directions. The forward LSTM produces hidden states $\vec{h_{t}}$, while the backward LSTM generates $\overset{⃖}{h_{t}}$. These two representations are concatenated to form the final hidden state $h_{t}=\left[\vec{h_{t}};\overset{⃖}{h_{t}}\right]$, where [;] indicates vector concatenation. Collectively, the hidden states form the matrix $H=\left[h_{1},h_{2},\ldots ,h_{T_{Seq}}\right]\in {\mathbb{R}}^{T\times 2h}$, with *h* representing the number of hidden units in a single LSTM direction and ℝ indicating real numbers.

The matrix *H* is then projected into Query, Key, and Value matrices defined as *Q* = *HW*_*Q*_, *K* = *HW*_*K*_, and *V* = *HW*_*V*_, where *W*_*Q*_, *W*_*K*_, and *W*_*V*_ are trainable weight parameters that are randomly selected based on the number of hidden neurons of the Bi-LSTM. The query, key, and value matrices enable adaptive weighting by employing the similarity measures between the features. This helps compute the self-attention scores, denoted as *S*_*Att*_, as given in Equation 8.


$$\begin{array}{c} S_{Att}=\text{softmax}\left(\frac{\left(HW_{Q}\right){\left(HW_{K}\right)}^{T}}{\sqrt{d_{k}}}\right) \end{array}$$ (8)

where $\sqrt{d_{k}}$ is the number of neurons that represent each query and key vector. The *Z*_*i*_ is the weighted combination of all the value vectors *V*_*j*_ indicated in Equation 9.


$$\begin{array}{c} Z_{i}=\overset{T}{\underset{j=1}{\sum }}S_{Att_{ij}}V_{j} \end{array}$$ (9)

where *i* is the entire sequence of the elements at *i*^*th*^ time step focus on *j*^*th*^ time step. Thus, the self-attention score (Z) is computed using Equation 10.


$$\begin{array}{c} Z=S_{Att}.V \end{array}$$ (10)

Thus, *Z* captures global dependencies among relevant features, enhancing their association and emphasizing informative patterns, leading to better classification.

## 4 Research model development using an empirical study

**Figure 2:**
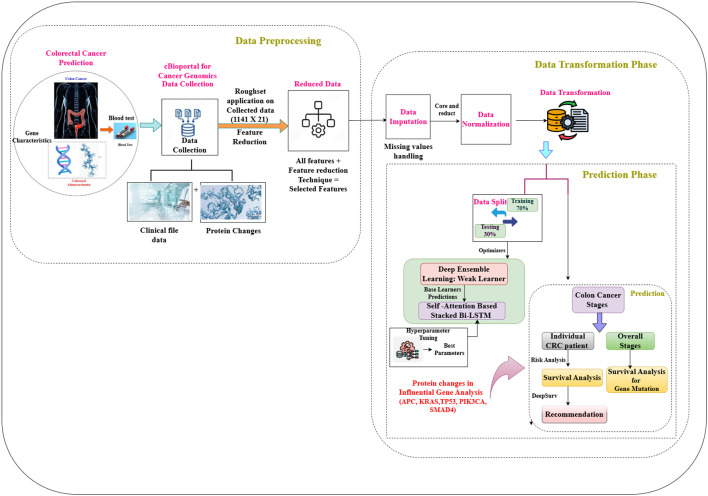
Proposed prediction model.

To demonstrate the research model development, a colorectal cancer dataset was collected from the cBio Cancer Genomics Portal (https://www.cbioportal.org/study/summary?id=crc_eo_2020%2Ccoadread_cass_2020) connected with the TCGA database repository. The collected CRC dataset consists of 1141 samples with 21 attributes for adenocarcinoma, including stage as the target (*d*). The collected data undergoes two phases, namely preprocessing and post-processing. In the preprocessing, the rough set data analysis is used for identifying the superfluous attributes, and in the postprocessing, the self-attention-based deep ensemble Bi-LSTM **(self-attention-RF-SBi-LSTM)** model, which optimizes the optimal parameters by using the Whale optimization algorithm (WOA) for global convergence, has been used to classify and predict the stages of colorectal cancer. The notation representation table is presented in Table 4. An abstract view of the proposed architecture is shown in Figure 2.

**Table 4:** Normalized information table.

Attributes	Notation	Normalized value	Classification
Age at diagnosis	[*a*_1_]	18–30	Low [1]
		31–60	Medium [2]
		61–80	High [3]
		81–93	Very High [4]
Gender	[*a*_2_]	—	Man [1]
			Woman [2]
BMI	[*a*_3_]	15.87–23.30	Low [1]
		23.31–26.50	Moderate [2]
		26.51–30.50	High [3]
		30.60–56.20	Very High [4]
Smoking history	[*a*_4_]	Never	Low [1]
		Ever	Medium [2]
		Former	High [3]
		Current	Very High [4]
Hypertension history	[*a*_5_]	No	Low [1]
		Yes	High [2]
Diabetes mellitus history	[*a*_6_]	No	Low [1]
		Yes	High [2]
Tumor grade	[*a*_7_]	Well-differentiated	Low [1]
		Moderately differentiated	Medium [2]
		Moderately to poorly differentiated	High [3]
		Poorly differentiated	Very High [4]
Tumor purity	[*a*_8_]	10%–25%	Low [1]
		25%–45%	Medium [2]
		45%–65%	High [3]
		65%–90%	Very High [4]
Primary tumor location	[*a*_9_]	Left colon	Low [1]
		Rectum	Medium [2]
		Right colon	High [3]
Fraction genome altered	[*a*_10_]	0.0001–0.05	Low [1]
		0.05–0.2	Medium [2]
		0.2–0.5	High [3]
		0.5–0.8235	Very high [4]
Somatic status	[*a*_11_]	Unmatched	Low [1]
		Matched	High [2]
Mutation count	[*a*_12_]	1–5	Low [1]
		6–19	Medium [2]
		20–50	High [3]
		51–7,006	Very high [4]
Protein change count	[*a*_13_]	APC(R876*, R1450*, T1556Nfs*)	Low [1]
		APC(R1450*), KRAS(G13A,D,C)	Medium [2]
		APC(R248Q*,..), TP53(R273,..)	High [3]
		APC, KRAS, PIK3CA (G364R, E542K, H10478), SMAD4 (R361 C,H)	Very high [4]
Tissue sample type	[*a*_14_]	Primary	Low [1]
		Local recurrence	Medium [2]
		Lymph node metastasis	High [3]
		Metastasis	Very High [4]
Impact TMB score	[*a*_15_]	0.1–10 mut/Mb	Low [1]
		10–20 mut/Mb	Medium [2]
		20–100 mut/Mb	High [3]
		100–368.6 mut/Mb	Very high [4]
MSI score	[*a*_16_]	–1–1	Low [1]
		1–10	Medium [2]
		10–30	High [3]
		30–48.45	Very high [4]
Metastatic site	[*a*_17_]	Liver	Low [1]
		Lung	Medium [2]
		Brain	High [3]
		Peritoneum	Very high [4]
Metastasectomy	[*a*_18_]	0	Low [1]
		1	Medium [2]
Overall survival (months)	[*a*_19_]	36.1–314.661 (good survival)	Low [1]
		12.1–36 (moderate survival)	Medium [2]
		6.1–12 (Poor survival)	High [3]
		0.2–6 (Very poor survival)	Very high [4]
Overall survival status	[*a*_20_]	Living	[1]
		Deceased	[2]
Stage at diagnosis	[*d*]	Stage I	[1]
		Stage II	[2]
		Stage III	[3]
		Stage IV	[4]

A better prediction process depends on analyzing the collected data. To ensure better results, the data should be consistent. However, the collected data contains missing values, leading to inconsistencies that need to be addressed. Data imputation is a widely used method for replacing missing values with the mean or median of the attribute values. The collected dataset contains 119 missing values, and mean imputation was applied to fill them. After imputation, each feature's standard deviation is assessed to assess the data's variability and gain insight into how much the imputed data deviates from the original distribution. On applying data imputation, it has been analyzed that there is no such deviation after imputation in the imputed features, as depicted in Table 5. Thus, the Roughset-based Core and Reduct algorithm is employed for feature selection to ensure data consistency and identify significant attributes.

**Table 5:** Data imputation for the missing values.

Features with	Count of missing values	Mean Imputation
BMI (*a*_3_)	51	27
Hypertension history (*a*_20_)	30	1
Tumor purity (*a*_8_)	22	2
Fraction genome altered (*a*_10_)	16	43

### 4.1 Roughset data reduction by applying core and reduct

This section describes the process of finding core and reduct attributes from the collected dataset. The concepts of core and reduct were previously explained in the Background Fundamentals section on Roughset-Core and Reduct. Based on this the indiscernibility relation was computed on sample of 10 patients and observed that the attributes Gender (*a*_2_), Smoking History (*a*_4_), Diabetes mellitus (*a*_6_), Metastatic site (*a*_18_) are identified as reducts, whereas the core attributes are Age(*a*_1_), BMI(*a*_3_), Hypertension(*a*_5_), Tumor Grade(*a*_7_), Tumor Purity(*a*_8_), Tumor Location(*a*_9_), Fraction Genome Altered(*a*_10_), Somatic Status(*a*_11_), Mutation Count(*a*_12_), Protein Change Count(*a*_13_), Tissue Sample Type(*a*_14_), TMB Score(*a*_15_), MSI Score(*a*_16_), Metastatic Site(*a*_17_), Overall Survival Months(*a*_19_), and Overall survival status (*a*_20_). The sample dataset for 10 patients is presented in Table 6 with 16 core attributes, and the normalized values are presented in Table 7. Thus, the superfluous attributes can be identified and removed without any information loss.


$$\begin{array}{c} \text{Core attributes}\\ =\left\{a_{1},a_{3},a_{5},a_{7},a_{8},a_{9},a_{10},a_{11},a_{12},a_{13},a_{14},a_{15},a_{16},a_{17},a_{19},a_{20}\right\} \end{array}$$

$$\begin{array}{c} \text{Reduct attributes}=\left\{a_{2},a_{4},a_{6},a_{18}\right\} \end{array}$$

**Table 6:** Sample dataset.

Patients	*a*_1_	*a*_3_	*a*_5_	*a*_7_	*a*_8_	*a*_9_	*a*_10_	*a*_11_	*a*_12_	*a*_13_	*a*_14_	*a*_15_	*a*_16_	*a*_17_	*a*_19_	*a*_20_	(*d*)
*p*_1_	56	27.81	No	Well-Diff	11	Leftcolon	0.2417	Unmatch	12	(APC, KRAS) = 2	Metastasis	18	0.67	Liver	35	Living	2
*p*_2_	67	28.24	No	Well-Diff	21	Left colon	0.4613	Matched	19	(APC PIK3CA) = 2	Metastasis	19	0.07	Liver	15	Living	2
*p*_3_	29	–	Yes	mod-diff	–	Rectum	0.3189	Matched	2764	(APC, KRAS, TP53, SMAD4) = 7	Lymph node Metastasis	315.6	27	'Lung	7	Living	3
*p*_4_	45	25.18	–	mod-poorly-diff	64	Right colon	0.0612	Matched	43	(APC, TP53) = 3	Primary	23	2	Lung	38	Living	1
*p*_5_	83	39.31	No	Poorly-diff	–	Rectum	–	UnMatched	34	(KRAS = 2)	Local Recurrence	9	43.27	Peritonm	3	Died	4
*p*_6_	52	24.39	Yes	mod-poorly-diff	86	Right colon	70.1984	UnMatched	13	(APC, PIK3CA) = 3	Primary	78	9	Lung	96	Living	3
*p*_7_	30	29.21	Yes	Well-diff	21	Left colon	0.0027	Matched	17	(APC, TP53, KRAS, SMAD4)= 11	Local Recurrence	354.3	39.45	Peritonm	8	Living	3
*p*_8_	81	42.99	No	Poorly-diff	63	Left Colon	0.7827	Matched	68.36	APC=3	Local Recurrence	8	46.83	Peritonm	4	Died	4
*p*_9_	72	19.39	No	mod-diff	39	Rectum	0.4629	Matched	4983	(KRAS, SMAD4)=3	Primary	125.7	27.3	Lymph node	15	Died	3
*p*_10_	25	16.24	Yes	mod-diff	42	Rectum	0.0034	Matched	6934	(APC, TP53, KRAS, SMAD4) = 9	Lymph node Metastasis	264.1	21.8	Lung	11	Living	3

**Table 7:** Normalized sample reduced dataset.

Patients	*a*_1_	*a*_3_	*a*_5_	*a*_7_	*a*_8_	*a*_9_	*a*_10_	*a*_11_	*a*_12_	*a*_13_	*a*_14_	*a*_15_	*a*_16_	*a*_17_	*a*_19_	*a*_20_	*d*
*p*_1_	3	3	1	1	1	1	3	1	2	1	4	2	1	2	2	1	2
*p*_2_	3	3	1	1	1	1	3	2	2	1	4	2	1	2	3	1	2
*p*_3_	1	2	2	2	2	2	3	2	4	3	3	4	3	1	3	1	3
*p*_4_	2	2	2	3	3	3	2	2	3	2	4	3	2	2	1	1	1
*p*_5_	4	4	1	4	3	2	4	1	2	4	1	1	4	3	3	2	4
*p*_6_	2	2	2	3	4	2	1	2	3	2	2	3	2	2	1	1	1
*p*_7_	1	3	2	1	1	3	1	2	2	4	2	4	4	1	3	1	3
*p*_8_	4	4	1	4	4	1	4	2	4	4	2	4	4	3	4	2	4
*p*_9_	3	1	1	2	2	2	3	2	4	3	3	4	3	2	2	2	2
*p*_10_	1	1	2	2	3	2	3	2	4	3	3	3	3	1	3	1	3

### 4.2 Postprocess architecture using self- attention based deep ensemble stacked Bi-LSTM

In post-processing, the consistent data of a total of 1,141 objects has class imbalance. To handle class imbalance, the SMOTE-Tomek technique is applied. After employing the Rough set core and reduct, the consistent dataset is split into 800 and 341 objects to preserve the original testing data for validation. The SMOTE-Tomek resampling method was applied to the training data, resulting in 2,764 samples, with 690 samples per decision class. The obtained objects were used for five-fold cross-validation with Random Forest (RF), Support Vector Machine (SVM), and Decision Tree (DT), and the accuracies were evaluated before and after applying SMOTE-Tomek. Furthermore, the Friedman statistical test was used to compare the performance of RF, SVM, and DT classifiers across the same cross-validation folds before and after applying SMOTE-Tomek. This non-parametric statistical test is used to evaluate multiple classifiers on collected data to address data leakage in resampled samples after SMOTE-Tomek. The obtained *P* = 0.0067 across all three classifiers indicates a statistically significant difference among them. Based on the average ranks, RF achieved the best rank (1.00) before and after SMOTE Tomek, with accuracies of 93.89% and 95.21%, compared to SVM and DT. After applying the feature reduction technique, 16 attributes were identified as core attributes, including survival variables, overall survival months (*a*_19_), and overall survival status (*a*_20_). So, these variables were included in the subsequent classification and survival prediction analyses due to their strong association with colorectal cancer progression and patient outcomes. Given the length of the paper, the cross-validation fold-wise accuracy results and the Friedman test ranking tables were not included.

**Figure 3:**
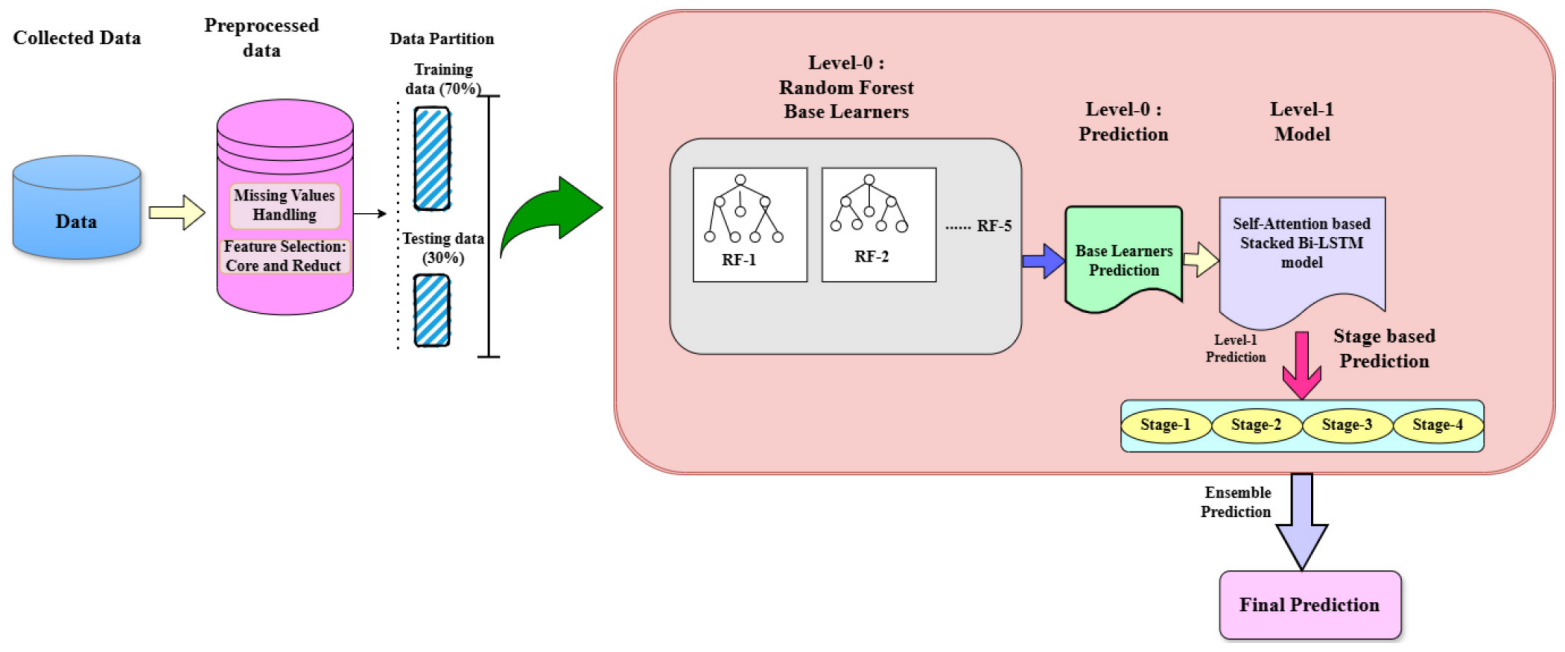
Classification and prediction with proposed deep ensemble learning model.

The improvement in accuracy after resampling the samples indicates significant data balancing and confirms that the random forest is robust to cross-validation. Thus, the data leakage was addressed, and the data was applied to the proposed methodology for classification and prediction. The training data is fed into the self-attention-based deep ensemble Bi-LSTM model, designed using the base and meta learners. Random Forest serves as a base learner, and Bi-directional Stacked LSTM as a meta-classifier, employing both static and sequential dependencies from the consistent data. The base learner captures diverse decision patterns across dimensional features, processed sequentially by a Stacked Bi-LSTM to learn temporal dynamics and hidden representations. Additionally, a self-attention layer is added to the Bi-LSTM to focus on the most relevant features within the sequence by learning the global feature interactions. Figure 3 represents the architecture of the proposed Self-attention based Stacked Bi-LSTM model, and the algorithm's working process is indicated in Algorithm 1.

Algorithm 1 Self-attention based deep ensemble stacked Bi-LSTM. 
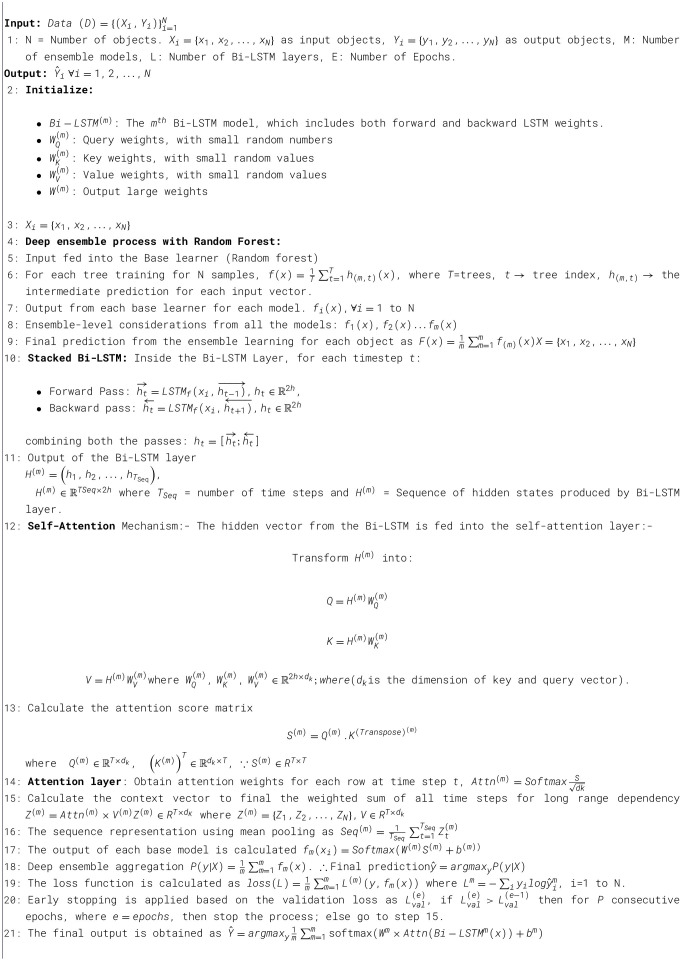

The random forest serves as the base learner for the five decision trees in the proposed model, with each tree trained on 160 training objects. Thus, the 800 training objects are trained in Level 0. The decision trees *T* in Level 0 independently learn patterns from the training data and produce an intermediate prediction denoted as *h*_(*m, t*)_(*x*). The predictions from all base models are combined as *f*_1_(*x*), *f*_2_(*x*)*f*_*m*_(*x*), where *m* is the total number of base models. Thus, the predictions of the base learner, indicated as *F*(*x*) for each input *x* ∈ *X* = {*x*_1_, *x*_2_, …, *x*_*N*_}, are computed by averaging the outputs of all *m* models and are now fed into the meta learner. The stacked Bi-LSTM layer acts as a meta learner; for each time step *t*, the sequence is processed in both directions, forward and backward. In the forward direction, the hidden state $\vec{h_{t}}$ is calculated using the current input and the previous forward hidden state. In the backward direction, the hidden state $\overset{⃖}{h_{t}}$ is computed using the current input and the next backward hidden state. The final hidden representation at time step t is obtained by combining the forward and backward hidden states as $\left(h_{t}=\vec{h_{t}}.\overset{⃖}{h_{t}}\right)$. The output of the Bi-LSTM layer is the sequence of hidden states $H^{\left(m\right)}=\left(h_{1},h_{2},\ldots ,h_{T_{\text{Seq}}}\right)$, Where *T*_*Seq*_ is the number of time steps.

The hidden vector from the Bi-LSTM is fed into the self-attention layer, thus transforming the *H*^(*m*)^ into Query (*Q*), Key (*K*), and Value (*V*). This transformation is performed using learnable weight matrices such as


$$\begin{array}{c} \;Q=H^{\left(m\right)}W_{Q}^{\left(m\right)} \end{array}$$

$$\begin{array}{c} \;K=H^{\left(m\right)}W_{K}^{\left(m\right)} \end{array}$$

$$\begin{array}{c} \;V=H^{\left(m\right)}W_{V}^{\left(m\right)} \end{array}$$

Where $W_{Q}^{\left(m\right)},W_{K}^{\left(m\right)},W_{V}^{\left(m\right)}\in {\mathbb{R}}^{2h\times d_{k}}$.

Furthermore, the attention score is computed by using the dot (.) product between the query and the transpose of the key matrix


$$\begin{array}{c} S^{\left(m\right)}=Q^{\left(m\right)}.K^{{\left(Transpose\right)}^{\left(m\right)}} \end{array}$$

which is fed into the attention layer to obtain the attention weights for each layer in the stacked Bi-LSTM at time step *t* in the ensemble process. The context vector is calculated to find the weighted sum of the time steps of the ensemble learning for the long-range dependencies indicated as


$$\begin{array}{c} Z^{\left(m\right)}=Attn^{\left(m\right)}\times V^{\left(m\right)}Z^{\left(m\right)}\in R^{T\times d_{K}} \end{array}$$

where *Z*^(*m*)^ represents the attention enhanced sequenced representation. The product of the weight matrix and the sequence matrix of each ensemble layer, added with the bias layer, enters the softmax layer to produce the aggregation of the deep ensemble layer indicated as *P*(*y*|*X*), and the final prediction is obtained as Ŷ. The loss function is used to determine the stopping criteria for the proposed model; thus, early stopping is applied to obtain the overall prediction.

#### 4.2.1 Global convergence using parallel-whale optimization algorithm

The Whale Optimization Algorithm (WOA) is a metaheuristic optimization technique inspired by nature, mimicking the bubble-net hunting behavior of humpback whales (solutions). The WOA finds globally optimal parameters by balancing exploration and exploitation through approaches such as shrinking encircling and spiral bubble-net feeding. The humpback whale uses random search for exploration and confined iterations for exploitation to avoid local optima. By adjusting the candidate positions within the search space toward the identified optimal solution (prey), the WOA algorithm continues until the termination condition is achieved.

Compared with traditional WOA, the proposed Parallel-WOA algorithm introduces a parallel fitness evaluation approach to reduce computational time for optimizing a computationally intensive deep learning model. Additionally, the algorithm is adapted to continuously tune the hyperparameters, enabling better optimization of the LSTM units, dropout rate, and learning rate. A boundary control mechanism was employed to ensure feasible solutions throughout the search space, and the convergence history was observed to assess the performance of the SA-RF-SBi-LSTM model. In parallel-WOA, each whale represents a candidate solution $X_{i}\in R^{d}$ within a defined search space. The algorithm begins by randomly initializing a population of whales within the given bounds and evaluates their fitness using the objective function *f*(*X*). At each iteration, *t* ∈ {1, 2, ..., *T*}, the better solution X^*^ is identified and is used for the further search process. The position of each whale is then updated using the encircling prey, exploration, and bubble-net attack. The encircling prey is configured as


$$\begin{array}{c} {\text{X}}_{i}^{t+1}={\text{X}}^{*}-A\cdot |\text{C}\cdot X^{*}-{\text{X}}_{i}^{t}| \end{array}$$

where, *A* = 2*ar*_1_ − *a, C* = 2*ar*_2_ and *r*_1_, *r*_2_ ∈ [0.1]. The control parameter *a* decreases from 2 to 0 by balancing exploration ad exploitation. When |*A* ≥ 1| a random whale *X*_*rand*_ is selected to enhance global exploration. In parallel, the bubble-net strategy updates positions using a spiral equation.


$$\begin{array}{c} {\text{X}}_{i}^{t+1}=|X^{*}-{\text{X}}_{i}^{t}|e^{bl}cos\left(2\pi l\right)+X^{*} \end{array}$$

where, *b* is a constant and *l* in [−1, 1]. In the proposed parallel-WOA algorithm, a multiprocessing pool is used to distribute fitness evaluation across multiple CPU cores, enabling parallel computation of objective function values and reducing computational time. The parallel-WOA algorithm iteratively refines whale positions until the maximum number of iterations is reached, thereby obtaining the optimal hyperparameters. The WOA search process with parallel fitness evaluation is presented in Algorithm 2.

Algorithm 2 Parallel-whale optimization algorithm for hyperparameter optimization. 
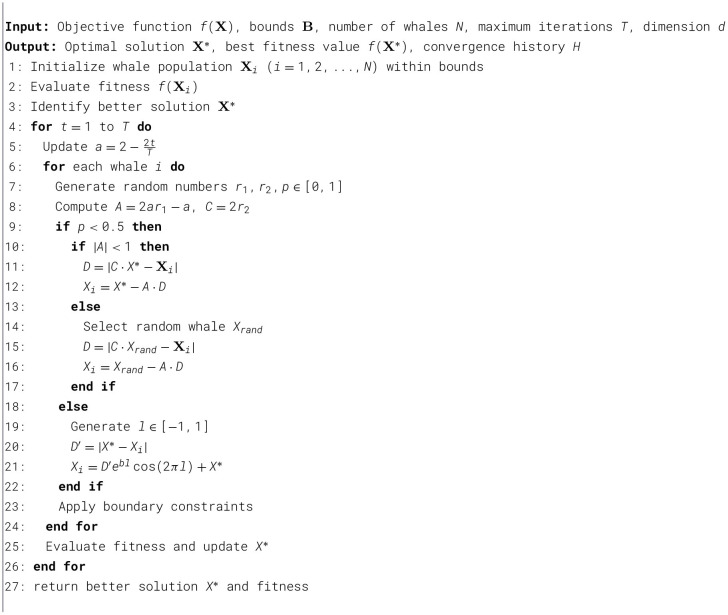

The proposed SA-RF-SBi-LSTM model was employed with parallel-WOA for hyperparameter tuning, where each candidate solution was evaluated using a stacked Bi-LSTM model with early stopping.

## 5 Experimental analysis and result discussion on stage-based CRC prediction

The experiments were conducted in Google Colab using Python 3.9 and TensorFlow/Keras. The model was trained and evaluated on standard computational hardware without reliance on specialized GPU acceleration. The experimental analysis was carried out in a Google Colab with a 12^*th*^ Intel i5 processor and 8GB RAM, Windows 11. To ensure reproducibility, experiments were repeated in five independent runs, and performance metrics such as accuracy, ROC-AUC, and F-score were reported as mean values with 95% confidence intervals. This setup demonstrates that the proposed workflow can be implemented on available resources. Thus, enhancing accessibility and reproducibility across different research environments. The consistent data of a total of **2,764** data objects undergoes classification by splitting the training data into **70% (2,764 objects)** and the testing data into **30% (341 objects)**. To enhance the predictive capability for implementing the self-attention-RF-SBi-LSTM (SA-RF-SBi-LSTM), the Parallel-Whale Optimization algorithm (WOA) was applied. In this study, the WOA was used to identify the optimal combination of LSTM units, dropout rate, and learning rate to achieve global convergence and improve model performance. Initially, a population of whales representing candidate hyperparameter solutions was randomly generated within predefined search ranges. Each whale is encoded as a potential parameter configuration, and its fitness is evaluated using the classification performance of the SA-RF-SBi-LSTM-WOA model. During each iteration, the whales updated their positions based on encircling prey, and bubble-net attacking (a random search mechanism) is enabled to balance between exploration of the search space.

To improve optimization, parallel processing using the Map() function is integrated. The map function enables concurrent evaluation of multiple candidate solutions and reduces the computational time required by the model's fitness function. For each candidate solution, the model was trained using the corresponding hyperparameters, and the resulting accuracy served as the evaluation criterion guiding the optimization process. Through the successive iterations, WOA progressively refined the search space and identified the best-performing hyperparameters. Now, the SA-RF-SBi-LSTM model is trained using the best hyperparameters identified by WOA. The hyperparameters for the implementation of Self-Attention-RF-SBi-LSTM-WOA are provided in Table 8.

**Table 8:** The tuning hyperparameters.

SA-RF-stacked bidirectional - LSTM - WOA			
Hyperparameters	Search space	WOA search Range	Selected Choice
Batch size	64, 32, 20	-	64
Data split	(60%–40%), (70%–30%), (80%–20%)	-	(70%–30%)
No. of units	(128, 64), (64, 32), (32, 16)	32–64	64
Dense layer units	32,64	-	64
Self-attention layer	Softmax pooling	-	
Number of epochs	5, 10, 15	-	5
Dropout rate	0.2, 0.3, 0.4, 0.5	0.2 - 0.5	0.2
Learning rate	0.001, 0.002, 0.005	0.0005–0.005	0.001
Optimizer	WOA, Adam, RMSprop, Nadam, SGD	-	WOA
No. of whales	-	5,6,8,10	10
No. of iterations	-	6,7,8,10	10

**Figure 4:**
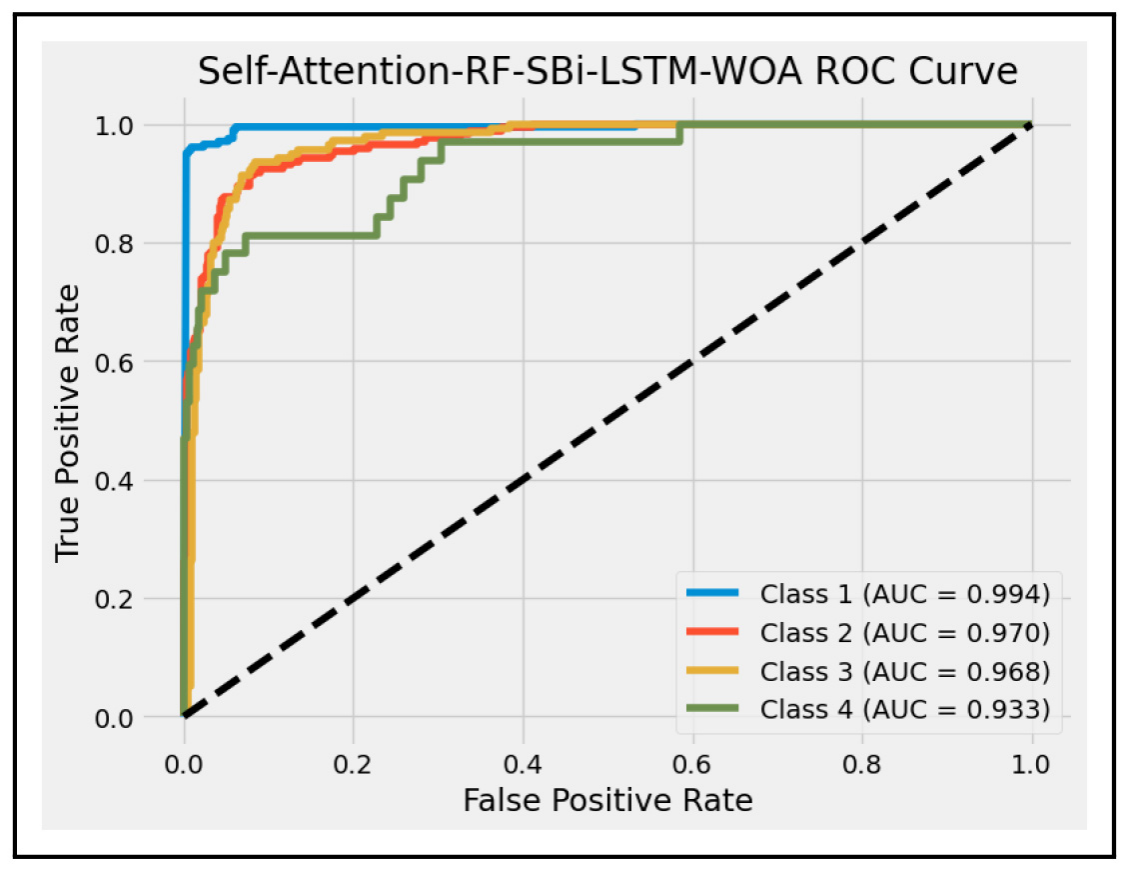
ROC-AUC curve of SA-RF-SBi-LSTM-WOA.

The preprocessed data was fed into both the Stacked Bi-LSTM with RF as base learner and the Self-Attention-based RF-SBi-LSTM (SA-RF-SBi-LSTM-WOA) model for training to obtain the classification accuracy. The training data was randomly divided into batches of 64, 32, and 20, and the batch size of 64 achieved higher accuracy than the others, as shown in Table 9. Furthermore, across the various optimizers considered in Table 8, the Whale optimizer achieves a higher accuracy of **96.78%** with batch size 64. The performance of the model is evaluated using confusion matrix metrics, where True Positives (TP), False Positives (FP), True Negatives (TN), and False Negatives (FN), indicated from Equations 11–16 and are represented in Table 10. The evaluation metrics, such as Precision, Recall, F-score, Specificity, Kappa score, KL Divergence, and ROC-AUC, are presented in Table 9 and shown in Figure 4. The calculation of metrics based on the classification of stages is as follows:

**Table 9:** Classification accuracy of evaluation metrics at early stopping.

	RF-SBi-LSTM			SA-RF-SBi-LSTM			SA-RF-SBi-LSTM-WOA		
Metrics	Batch size-64	Batch size-32	Batch size-20	Batch size-64	Batch size-32	Batch size-20	Batch size-64	Batch size-32	Batch size-20
Precision	89.26	85.24	65.73	92.02	88.26	69.36	**92.98**	89.97	70.42
Recall	90.37	85.34	66.72	79.02	92.68	70.69	**93.56**	80.54	71.58
Specificity	83.75	82.32	62.89	90.43	76.45	69.59	**91.52**	78.59	70.84
F-Score	71.45	70.32	61.97	92.76	75.24	70.56	**93.65**	76.53	71.98
Accuracy	81.32	79.86	77.12	92.85	89.91	84.10	**96.78**	89.78	95.34
Kappa Score	0.69245	0.68215	0.66342	0.92113	0.82261	0.70657	**0.93124**	0.83262	0.77852
KL-Divergence	0.90478	0.89734	0.69762	0.95804	0.72678	0.70632	**0.96502**	0.74791	0.71896
ROC-AUC-Score	0.88547	0.86765	0.79381	0.95605	0.92824	0.66374	**0.97607**	0.93982	0.68378

The obtained results of evaluation metrics of proposed SA-RF-SBi-LSTM-WOA are indicated in bold.

**Table 10:** Confusion matrix of SA-RF-SBi-LSTM-WOA.

Actual/ predicted	Stage 1	Stage 2	Stage 3	Stage 4
Stage 1	672	17	2	0
Stage 2	10	670	11	0
Stage 3	0	0	673	6
Stage 4	0	0	31	660

The Precision(Prc) indicates the model's accuracy in predicting positive stage predictions:


$$\begin{array}{c} Prc=\frac{TP}{TP+FP} \end{array}$$ (11)

The Recall(Rec) measures the ability to identify true positives from the total number of actual positives, which is important for clinical data:


$$\begin{array}{c} Rec=\frac{TP}{TP+FN} \end{array}$$ (12)

The F-score(Fscr) balances the harmonic mean of precision and recall to provide a single metric for performance:


$$\begin{array}{c} Fscr=\frac{2\left(Precision*Recall\right)}{\left(Precision+Recall\right)} \end{array}$$ (13)

The Specificity(Spc) measures the ability of the model to identify true negatives, which is particularly important in minimizing false positive predictions in clinical diagnoses:


$$\begin{array}{c} Spc=\frac{TN}{TN+FP} \end{array}$$ (14)

Cohen's Kappa score:


$$\begin{array}{c} k_{c}=\frac{P_{o}-P_{e}}{1-P_{e}} \end{array}$$ (15)

Where *P*_0_ = Observed proportion of the agreement between rating; *P*_*e*_ = Expected proportion of agreement between rating; and the value of the kappa score ranges from -1 (worse than perfect chance) and 1 (better agreement).

K.L Divergence (Kullback-Liebler Divergence)


$$\begin{array}{c} D_{KL}\left(P∥Q\right)=\underset{i}{\sum }P\left(i\right)\log\frac{P\left(i\right)}{Q\left(i\right)} \end{array}$$ (16)

Where *P*(*i*) and *Q*(*i*) are the first and second distributions of event *i* with natural log logarithm.

Table 11 shows that the evaluation metrics Prc, Rec, Spec, Fscr, and ACC for **SA-RF-SBi-LSTM-WOA** model have values of **92.98, 93.56, 91.52, 93.65** and **96.78** respectively. The model was trained, and validation was carried out using the confusion matrix on the training data, along with the ROC curve and the corresponding AUCs. Figure 4, the ROC-AUC curve for the proposed SA-RF-SBi-LSTM-WOA model illustrates the difference between four different classes [Class 1 (Stage 1), Class 2 (Stage 2), Class 3 (Stage 3), and Class 4 (Stage 4)].

**Table 11:** Mean performance and confidence interval.

Metric	Mean performance	95% confidence interval	
Accuracy	96.78%	[95.92%–97.64%]	Stable across 5 independent runs
ROC-AUC score	0.97%	[0.96%–0.98%]	Consistent values
F-score	0.93%	[0.92%–0.96%]	Consistent values
Precision	0.92%	[0.92%–0.94%]	Consistent values
Recall	0.93%	[0.92%–0.94%]	Consistent values

Per class predictions for the proposed SA-RF-SBi-LSTM-WOA model are as follows:

- Stage 1: Represents the better classification of the model with an AUC of 0.994.
- Stage 2: Indicates a descent performance with an AUC of 0.970.
- Stage 3: Shows good performance with an AUC of 0.968.
- Stage 4: Indicates a fairly accurate performance with an AUC of 0.933.

The robustness analysis shows that the reported classification accuracy of 96.78% was consistent; we recognize the importance of ensuring robustness and reproducibility. To this end, all experiments were repeated across five independent runs, and the mean performance, along with 95% confidence intervals (95.92%–97.64%), was reported. Similarly, the confidence intervals for ROC-AUC, F-Score, Precision, and Recall provide statistical assurance of the model's stability and reliability. Also, the balance between precision and recall was stable. Furthermore, early stopping was employed during training by halting optimization once validation performance became stable. This design minimizes the risk of overfitting and ensures that the reported accuracy reflects consistent performance rather than a single favorable run. The Table 11 provides confidence intervals for validation metrics.

Based on the classification accuracy obtained using consistent data and the performance analysis, the proposed model performs better with a batch size of 64 for prediction accuracy. The stage-based prediction of the test objects achieves 84.75% accuracy for the RF-SBi-LSTM model and **90.02%** for the Self-Attention-RF-SBi-LSTM-WOA model. An ablation study of each model contribution of the proposed study is presented in Table 12. Thus, The prediction accuracy is evaluated on the remaining 341 test objects, as listed in Table 13.

**Table 12:** Ablation study of model contribution.

Model	Accuracy	F1-score	Precision	Recall	ROC-AUC score	Contribution
Random forest (RF)	0.80	0.70	0.87	0.88	0.86453	A base learner to capture the diverse decision pattern across the dimensional features
RF-Stacked Bi-LSTM	0.81	0.71	0.89	0.90	0.88547	Bi-directional context improves sequential
(SBi-LSTM)						learning
RF- Self attention (SA)	0.92	0.92	0.92	0.79	0.92825	Highlights influential
SBi-LSTM						genomic signals
Whale optimization	0.96	0.93	0.92	0.93	0.97607	Efficient hyperparameter
RF-SA-SBi-LSTM						tuning enhances
(Proposed model)						generalization

**Table 13:** Predicion accuracy of proposed models.

No.of objects	RF-SBi-LSTM model	SA-RF-SBi-LSTM model	SA-RF-SBi-LSTM-WOA model
42	33 (78.57%)	35 (83.34%)	36 (85.71%)
84	68 (80.95%)	71 (84.52%)	73 (86.90%)
126	103 (81.74%)	107 (84.92%)	110 (87.31%)
168	138 (82.14%)	143 (85.12%)	147 (87.50%)
210	174 (82.85%)	180 (85.71%)	185 (88.09%)
252	210 (83.33%)	217 (86.12%)	224 (88.89%)
294	247 (84.01%)	255 (86.73%)	263 (89.45%)
341	289 (84.75%)	298 (87.39%)	**307 (90.02%)**

The predicted accuracy and objects of proposed SA-RF-SBi-LSTM-WOA are indicated in bold.

## 6 Comparative analysis with existing benchmark models

**Figure 5:**
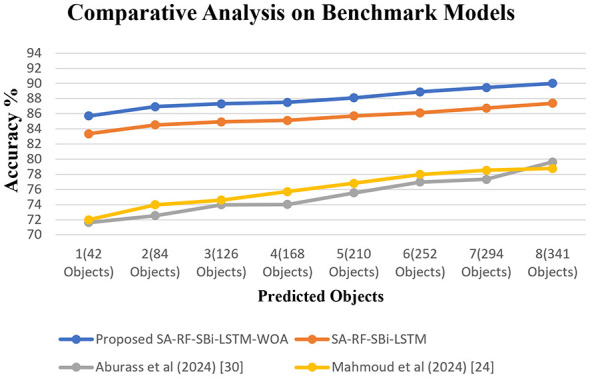
Comparative analysis of the benchmark model with the proposed SA-RF-SBi-LSTM-WOA model.

A comparative analysis has been carried out with the proposed model, SA-RF-SBi-LSTM-WOA, which is compared with benchmark models by Mahmoud et al. (2024) and Aburass et al. (2024), a genomic prediction for colorectal cancer with parameters such as execution time, accuracy, and value loss as represented in Table 14. The proposed model used early stopping to monitor performance on a validation set and terminate training when the validation loss no longer improved. The proposed model achieved optimal performance at epoch 5, achieving a prediction accuracy of **90.02%**, a validation loss of 0.0011, and an execution time of 16 min. Comparatively, the benchmarked models achieved extended execution times of 7–9 epochs, with higher validation loss and lower accuracy than the proposed model. Table 14 shows that the Attention-driven RF-SBi-LSTM-WOA model outperforms the benchmarked model, as demonstrated by the prediction analysis. From Figure 5 and Table 14, the proposed Self-Attention-driven RF-SBi-LSTM model achieves the highest accuracy of **90.02%** compared to benchmarked models. Furthermore, Table 14 provides the execution times for a batch size of 64 for **SA-RF-SBi-LSTM-WOA** and the benchmarked models.

**Table 14:** Execution time with prediction accuracy.

Parameters	SA-RF-SBi-LSTM-WOA	Mahmoud et al. (2024)	Aburass et al. (2024)
	Prediction	Prediction	Prediction
Accuracy (%)	87.50	76.91	70.03
Value-loss	0.0153	0.0232	0.0247
Epochs	1	3	4
Exec.time (mins)	7	14	21
Accuracy (%)	88.09	77.21	70.22
Value-loss	0.0115	0.0210	0.0229
Epochs	2	4	5
Exec.time (mins)	9	16	25
Accuracy (%)	88.89	77.89	70.91
Value-loss	0.0078	0.0195	0.0201
Epochs	3	5	7
Exec. time (mins)	12	20	32
Accuracy (%)	89.45	78.39	72.64
Value-loss	0.0062	0.0185	0.0176
Epochs	4	7	8
Exec.time (mins)	14	20	38
Accuracy (%)	**90.02**	79.76	74.62
Value-loss	**0.0011**	0.0173	0.0156
Epochs	**5**	8	9
Exec.time (mins)	**16**	30	45

The execution time with prediction accuracy are indicated in bold.

The RF-SA-SBi-LSTM-WOA model performs better when training with an increased batch size, and execution time increases from 5, 8, 10, 12 to 14 min. The model achieved increased execution times of 7, 10, 14, 16, and 18 mins with batch sizes of 10, 32, and 64 for prediction (testing data). The Self-Attention-RF-SBi-LSTM-WOA model performed better in terms of execution time on both training and testing data than other models. However, the proposed deep ensemble model has some limitations: it incurs computational overhead due to iterative hyperparameter optimization, which may lead to overfitting when trained on limited datasets. The computational burden was addressed by using a parallel fitness evaluation approach, enabling concurrent computation across multiple CPU cores. Thus, the model achieved a better execution time.

## 7 Survival analysis phase with case study

This section discusses the survival analysis of patients by identifying the influencing genes, including TMB and MSI scores, from the collected dataset. Key driver genes such as APC, KRAS, TP53, PIK3CA, and SMAD4 are widely recognized for their roles in colorectal cancer (CRC) initiation and progression, and their mutation patterns provide significant insights into disease severity and patient prognosis. The dataset comprises patients with more than two mutations in these influential genes, indicating greater genomic instability. Such multiple mutations are strongly associated with aggressive tumor behavior and variations in treatment response. The inclusion of TMB and MSI scores further strengthens the analysis, as high TMB reflects an increased number of mutations, while MSI status indicates defects in DNA mismatch repair mechanisms—both of which are critical biomarkers in cancer survival studies.

Table 15 highlights the unique protein alterations that significantly contribute to colorectal cancer (CRC) stage progression across five key genes: APC, KRAS, TP53, PIK3CA, and SMAD4. The**APC** mutation R1450* (a nonsense mutation) disrupts its tumor-suppressor function, leading to β-catenin accumulation and uncontrolled cell proliferation, particularly in early stages (Zhang et al., 2024; Sheng et al., 2024). **KRAS mutations** such as G13D are observed across all stages, while additional variants like G13C and Q61K increase tumor aggressiveness and metastatic potential. **TP53** mutations impair DNA repair mechanisms; early mutations (R175H, C176Y, and E224D) contribute to tumor heterogeneity, whereas advanced-stage mutations (C124Af46, C124R, C141Y, and E271) are associated with rapid tumor growth, genetic instability, and poor differentiation. **PIK3CA** mutations (G364R and H1047R) activate the PI3K/AKT pathway, promoting proliferation from Stage 1 to Stage 3, with additional mutations like Q546R influencing prognosis in Stage 4.

**Table 15:** Stage-wise identified unique protein changes contributed to stage progression in influential genes.

Protein changes					
Stages of CRC	APC	KRAS	TP53	PIK3CA	SMAD4
Stage-1	S1415Rfs*4/R564*/	R68S/G12S/R1114*	T1052K/**R175H**	**G364R**/H1047L	D537E/R361C
	X470_splice, T1388Yfs*7	D33E/G12_M16del	V157F/V173L	Q546K/T1052K	C115Y/G365D
	D777Kfs*8/R213*	G365D/G510R			
Stage-2	A2008V/R904Kfs*7/	Q61H/Q61P	R175H/**C176Y**/	H1047R/D1018del/	S232Qfs*3/Q534P
	Q1406Wfs*2/E1132*/	A146T/Q22K	E224D/V173L	P34L/R164Q	A327V/D351G/D537E
	A1446Vfs*27		L145R/P151Rfs*27		
Stage-3	A1283*/A1492Pfs*15/	**G13C**/Q61R	C124Afs*46/C124R/	D350G/D725G/	A532D/D355G
	E1284**/E1306*/	**G13D**/A146T/**Q61K**	**C141Y**/D259Y/E271*	E365K/E453_L455del	D537H/G231Afs*10
	K1053Nfs*3/K581Rfs*9/	/**H1047R**			
	M1431Cfs*42	E13090fs*4		V437D	
Stage-4	A1296Qfs*9/A1351Gfs*3/	L19F/Q61P	A161T/C124G	**Q546R**/R93W	A36_K45del/D52Rfs*2
	A703E/C1387*/R876*/R1450*	Q61L/G12V	K132E/P27Lfs*17	D350N/E542A	G352R/G419A
	/T1556Nfs*3/D1486Ifs*21				G419V/K436N
	/M743Cfs*18/L1382Tfs*4				

Unique protein changes are indicated as bold. ^*^indicates a non-sense mutation such as stop codon.

From the collected data, a significant number of multi-gene co-mutations were identified among patients with mutations, particularly in APC, KRAS, TP53, PIK3CA, and SMAD4. Triple and quintuple co-mutation patterns are prevalent, and increased genomic instability indicates the disease progression (He et al., 2025). Table 16 illustrates the gene combinations with the number of patients. Among these, APC shows the highest driver mutation rate (75.7%), followed by TP53 (73.1%) and KRAS (42.8%). Conversely, PIK3CA (18.8%) and SMAD4 (15.1%) exhibit lower mutation frequencies. These findings indicate that APC and TP53 mutations are more prominent in colorectal cancer progression than those in other genes. The genomic markers identified in this study, such as APC, KRAS, TP53, and SMAD4, represent critical drivers of colorectal cancer progression. Table 17 provides the biological significance of the identified genomic markers.

**Table 16:** Co-occurring mutations of 5 genes.

Co-occurring mutations of 5 genes	
Gene combination	Number of patients
APC + TP53	456
APC + KRAS + TP53	242
APC + KRAS	90
APC + KRAS + PIK3CA	70
APC + TP53 + PIK3CA	57
APC + KRAS + TP53 + PIK3CA	41

**Table 17:** Biological significance of the identified genomic markers.

Gene	Biological role in CRC progression	Clinical relevance
APC	Tumor suppressor; loss of function disrupts Wnt/β-catenin signaling, initiating adenoma formation.	Early driver mutation; predictive of adenoma-carcinoma sequence; informs screening strategies
KRAS	Oncogene: activating mutations stimulate the MAPK/ERK pathway, driving proliferation and therapy resistance.	Associated with poor response to anti-EFGR therapies; prognostic marker for aggressive disease.
PIK3CA	Oncogene; mutations activate the PI3K/AKT pathway, altering metabolism and promoting tumor progression.	Linked to resistance to targeted therapies; potential biomarker for PI3K inhibitors.
TP53	Tumor suppressor; mutations impair DNA damage response and apoptosis, fostering genomic instability.	Associated with poor prognosis and advanced disease; marker of aggressive phenotypes.
SMAD4	Tumor suppressor; Loss disrupts TGF- signaling by reducing growth inhibition and enhancing invasion.	Correlates with metastatic potential and poor survival; prognostic biomarker in advanced CRC.

### 7.1 Stage-based survival analysis using DeepSurv

The study applied a deep learning-based DeepSurv model for survival analysis to predict the survival probability of CRC patients using selected features, including BMI, TMB score, Protein change count, Overall survival months, Mutation count, and influential genes. The DeepSurv model shows better predictive performance by handling the censored data and capturing the interactions between genomic features using the hazard function as shown in Equation 17.

The DeepSurv models the hazard function


$$\begin{array}{c} h\left(t|x\right)as:h\left(t|x\right)=h_{0}\left(t\right)\cdot \exp\left(g\left(x\right)\right) \end{array}$$ (17)

where *h*_0_(*t*) is the baseline hazard and *g*(*x*) is a neural network output trained to capture non-linear covariate effects.

**Figure 6:**
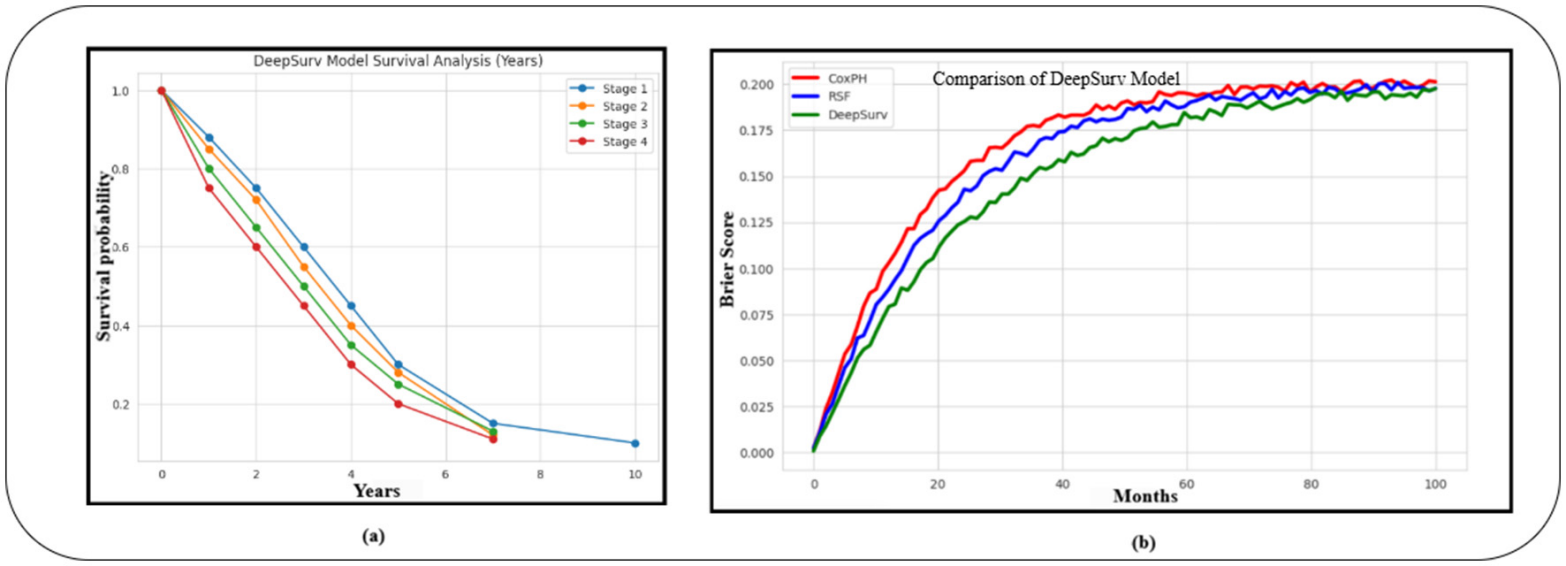
**(a)** 10-year survival analysis of the DeepSurv model in stages. **(b)** Comparison of Survival Analysis using DeepSurv Vs Cox and RSF.

The DeepSurv model is implemented in Google Colab version 3.0 with a vanilla MLP classifier. The DeepSurv model's performance was evaluated using the key metric, the Integrated Brier Score (IB-Score). Figure 6 depicts the overall survival analysis of CRC patients. Figure 6a shows the 10-year survival analysis of the DeepSurv model in stages, while Figure 6b compares the DeepSurv model with traditional survival analysis models like Cox proportional hazards (Cox) and Random Survival Forest (RSF).

**Figure 7:**
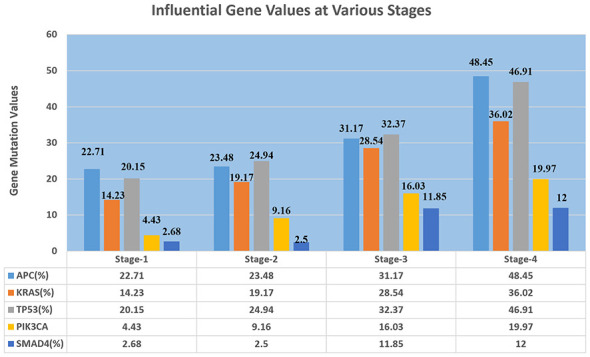
Influential gene values at various stages.

**Figure 8:**
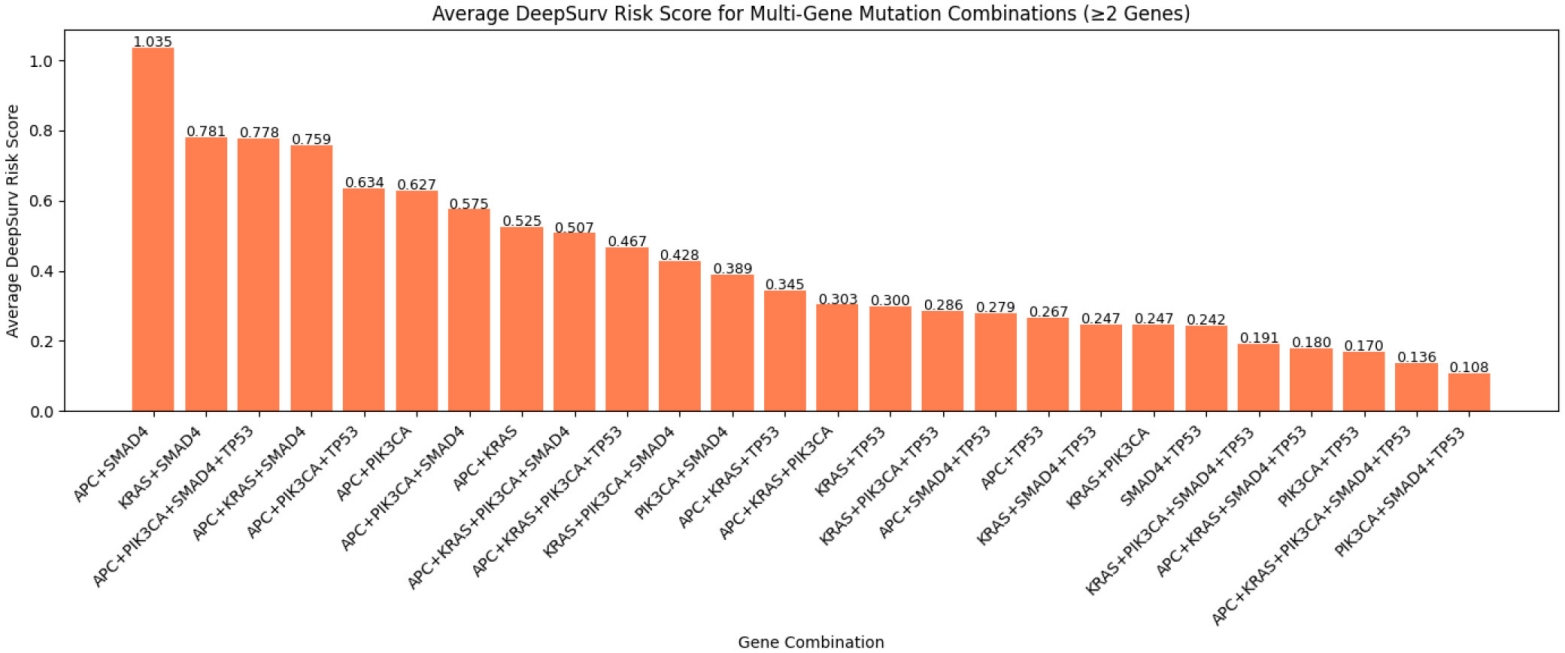
DeepSurv risk scores for co-occurring gene mutations in colorectal cancer.

Table 18 shows the hazard ratios of biomarkers for the 10-year prediction of survival with the DeepSurv model, including integrated Brier score (IB- score), hazard ratio, Partial Log-likelihood ratios, Concordance Index (C-index), 95% confidence intervals, and time-dependent AUCs for 10years. The DeepSurv model outperformed other survival analysis models. The IB-scores of DeepSurv are lower than those of other survival models, indicating that DeepSurv has predicted better risk stratification for the study. Figure 7 shows the influential gene mutation values at various stages. Figure 8 shows the Deepsurv risk scores for co-occurrence gene mutations.

**Table 18:** Ten-year statistical survival analysis of the DeepSurv model with other models.

Survival analysis models	Validation metrics			95% confidence intervals (CI)			
	IB score	Log-likelihood ratio	C-index	Hazard ratio	3-years AUC (%)	5-years AUC (%)	10-years AUC (%)
COX	0.166293	892.47	0.79	0.894	0.8277%	0.7616%	0.7507%
				[CI:0.87–0.91]	[CI:0.79–0.84]	[CI: 0.72–0.80]	[CI: 0.74–0.80]
RSF	0.159556	743.52	0.72	0.763	0.8158%	0.7267%	0.6946%
				[CI:0.74 - 0.80]	[CI:0.79 - 0.84]	[CI:0.69 – 0.74]	[CI:0.67–0.70]
Deep Surv	0.140072	916.24	0.82	2.120	0.9188%	0.8984%	0.8378%
				[CI:2.0–2.3]	[CI:0.89–0.94]	[CI:0.87–0.90]	[CI:0.79–0.85]

### 7.2 Case study

**Figure 9:**
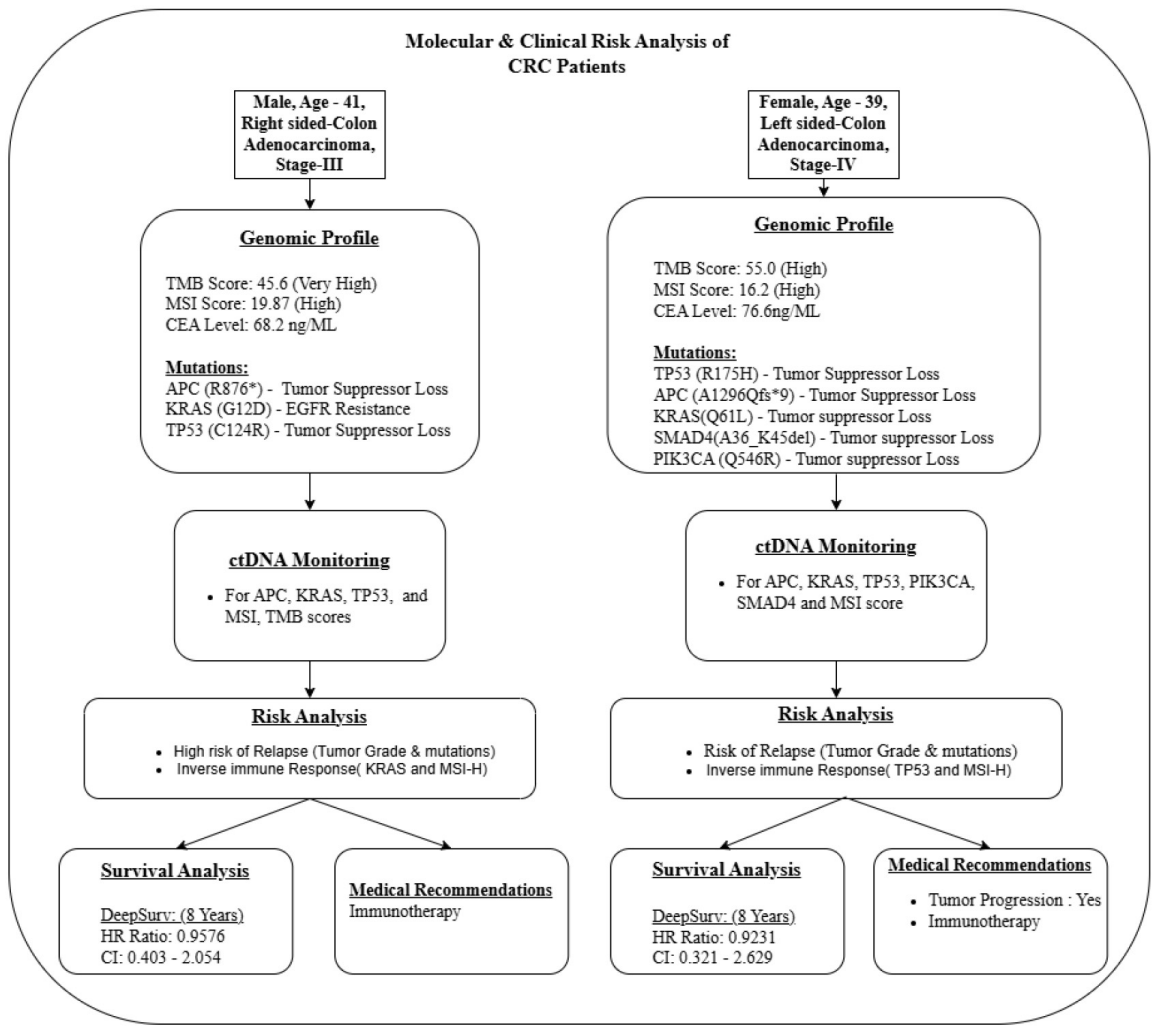
Risk analysis and prediction of CRC patients.

This section presents a case study on the comparative risk analysis of two colorectal cancer patients, integrating clinical stage information with gene mutation profiles and tumor biomarkers for each patient, as shown in Figure 9. In the first case, a 41-year-old man with stage III right-sided colon adenocarcinoma exhibits a very high tumor mutational burden (TMB), high microsatellite instability (MSI), and mutations in APC, KRAS, and TP53 genes. Similarly, the second case involves a 39-year-old woman with stage IV left-sided colon adenocarcinoma, showing high TMB, high MSI, and additional mutations including PIK3CA and SMAD4. These genomic features, along with circulating tumor DNA (ctDNA) monitoring, are fed into the DeepSurv model to estimate survival probabilities and relapse risks. DeepSurv integrates these multidimensional inputs to identify non-linear relationships between genetic mutations and patient outcomes, providing hazard ratios and confidence intervals for survival analysis. In both cases, the model predicts a high risk of relapse due to tumor grade and mutation burden, while also highlighting immune response variations linked to MSI status. Importantly, DeepSurv aids clinicians in understanding disease progression from stage III to stage IV by quantifying how genetic alterations accelerate risk, thereby guiding treatment decisions such as immunotherapy. Overall, the model enhances precision oncology by offering data-driven insights into CRC progression, enabling early intervention and improved patient-specific management strategies. By analyzing patients at different stages of colorectal cancer, the study illustrates how genetic mutations contribute to variations in tumor aggressiveness, prognosis, and potential treatment. The survival scores are obtained for men with an HR Ratio (0.9576) and women with an HR Ratio (0.9231).

#### 7.2.1 Clinical and technical contributions

- **Mutation insights for personalized medicine:** The proposed study highlights the significance of mutation insights, including APC, KRAS, TP53, PIK3CA, and SMAD4 mutations in customizing possible treatments for colorectal cancer patients.
- **Deep ensemble model:** The integration of five random forest classifiers as base learners with a self-attention-based stacked Bi-directional LSTM as the meta-classifier, optimized by the WOA optimization algorithm (WOA), highlights the efficiency and reliable prediction performance.
- **Risk stratification:** The learned hazard ratio helps stratify patient categories into high- and low-risk categories, guiding therapy decisions. By visualizing mutated residue positions, clinicians can gain actionable insights into how specific alterations (e.g., TP53 hotspots) correlate with survival outcomes.

## 8 Conclusion with future enhancement

The proposed study uses a deep ensemble model, RF-SBi-LSTM, with a Self-Attention layer for stage-based classification and survival analysis with Colorectal cancer data. The Rough Set-based Core and Reduct are used for feature selection and data consistency. The consistent data is fed into the deep ensemble model for classification and prediction. The proposed research is evaluated against benchmarking techniques to assess its performance. Furthermore, gene mutations are examined across various stages to assess the impact of each gene on cancer progression. Survival predictions are stratified using the DeepSurv model for clinical decision support. The evaluation on an independent dataset provides stronger evidence of the model's generalizability and clinical applicability. In the present study, the analyses were conducted using the available dataset, and model performance was assessed through rigorous internal validation procedures, including cross-validation. These procedures were adopted to minimize overfitting and obtain an unbiased estimate of predictive performance. However, an independent external dataset with comparable genomic characteristics and annotation standards was not available for this study. We acknowledge that the absence of external validation represents a limitation of the study and have explicitly stated this in the revised manuscript. Future studies will focus on validating the proposed framework using publicly available genomic datasets to assess its robustness, reproducibility, and generalizability across diverse patient populations. Future enhancements may involve the integration of genomic data (protein-protein Interaction (PPI) networks, particularly focusing on phosphatidylinositol phosphate (PIP) signaling), which shows uncovered new ways that the body regulates itself and responds to immune challenges, multi-modal data fusion, and the incorporation of explainable AI to enhance model interpretability with multi-objective optimization.

## Data Availability

The original contributions presented in the study are included in the article/supplementary material, further inquiries can be directed to the corresponding author.
